# Migrant-friendly hospitals: a paediatric perspective - improving hospital care for migrant children

**DOI:** 10.1186/1472-6963-13-389

**Published:** 2013-10-05

**Authors:** Fabienne N Jaeger, Ligia Kiss, Mazeda Hossain, Cathy Zimmerman

**Affiliations:** 1 London School of Hygiene and Tropical Medicine (LSHTM), Keppel street, London WC1E 7HT, UK; 2 Currently: Swiss Tropical and Public Health Institute, Socinstrasse 57, 4051 Basel, Switzerland; 3 Currently: University of Basel, Basel, Switzerland

**Keywords:** Migrant, Migrant-friendly, Hospital, Service delivery, Foreign, Child, Paediatric, Pediatric, Switzerland

## Abstract

**Background:**

The European Union (EU) Migrant-Friendly Hospital (MFH) Initiative, introduced in 2002, promotes the adoption of care approaches adapted to meet the service needs of migrants. However, for paediatric hospitals, no specific recommendations have been offered for MFH care for children. Using the Swiss MFH project as a case study, this paper aims to identify hospital-based care needs of paediatric migrants (PMs) and good service approaches.

**Methods:**

Semi-structured interviews were conducted with principal project leaders of five paediatric hospitals participating in the Swiss MFH project. A review of the international literature on non-clinical hospital service needs and service responses of paediatric MFHs was conducted.

**Results:**

Paediatric care can be complex, usually involving both the patient and the patient’s family. Key challenges include differing levels of acculturation between parents and children; language barriers; cultural differences between patient and provider; and time constraints. Current service and infrastructural responses include interpretation services for PMs and parents, translated information material, and special adaptations to ensure privacy, e.g., during breastfeeding. Clear standards for paediatric migrant-friendly hospitals (P-MFH) are lacking.

**Conclusions:**

International research on hospital care for migrant children is scarce. The needs of paediatric migrants and their families may differ from guidance for adults. Paediatric migrant needs should be systematically identified and used to inform paediatric hospital care approaches. Hospital processes from admission to discharge should be revised to ensure implementation of migrant-sensitive approaches suitable for children. Staff should receive adequate support, such as training, easily available interpreters and sufficient consultation time, to be able to provide migrant-friendly paediatric services. The involvement of migrant groups may be helpful. Improving the quality of care for PMs at both policy and service levels is an investment in the future that will benefit native and migrant families.

## Background

In a world of growing population mobility, many health providers and policy-makers have begun to consider the diverse care needs of migrant populations [1-3]. In 2002, the European Commission launched the European Migrant Friendly Hospital (MFH) Initiative, which fosters collaborations between hospitals, experts and non-governmental organisations (NGOs) in twelve European countries to develop migrant-friendly, culturally competent hospital care [4]. While encouraging greater attention to the healthcare needs of migrants, these efforts have fostered very limited dialogue and planning for the particular care approaches required for migrant youth or paediatric migrants (PM). This study focused on Switzerland’s MFH providers to explore policies, care approaches and potential adaptations required to meet the health and service needs of paediatric migrants [5].

### Migrant-friendly hospitals

At the start of the European MFH initiative, the scientific co-ordinating institution of the project (Ludwig Boltzmann Institute for the Sociology of Health and Medicine, Vienna) in collaboration with international experts and the partner hospitals, conducted a needs assessment and reviewed potentially effective interventions [6] to improve the responsiveness of hospital care to migrant patients’ needs. The following priorities for improvement were identified: 1) interpreting services; 2) staff training to increase cultural competence; and 3) patient information and parental training for mother and child care. Additional priority needs identified were adequate food and nutrition as well as spiritual and social support [4].

The resulting Amsterdam Declaration towards MFHs in an ethno-culturally diverse Europe [7] was launched, which offered broad recommendations on MFH care. An MFH task force established within the World Health Organisation (WHO) Network for Health Promoting Hospitals (HPH) [4] is currently developing standards [8]. To our knowledge, the development of child care-specific recommendations still needs to be further developed, as we could not find child health care-specific recommendations.

### A case study: Switzerland and MFH

Switzerland, which is not an EU member state, is currently promoting an MFH project with various paediatric clinics to further increase the ‘migrant-friendliness’ of its facilities.

The “Strategy Migration and Health 2002-2007” funded a network of 25 hospitals to participate in MFH care projects [9], networking with WHO Health-Promoting-Hospitals. The resulting book “Diversity and Equality of Opportunity” [1] was intended to guide hospitals in their efforts to become more migrant friendly [10]. See Table 1 for an overview of recommendations. Of 25 projects, only one has focused on paediatrics (particularly, paediatric interpreting services) [11].

**Table 1:** **Recommendations summary from “Diversity and Equality of Opportunity” Saladin, P et al.**[1]

**Topic**	**Summary recommendations**
Basic structure of organisation	Develop organisational policy to take account of migrant issues, including in mission, strategy, portfolio of services, resources.
	Ensure committed management
Migrant-specific data	Collect data specific to migrants and disaggregate migrant data
Structure, Processes & Outcome Quality	Ensure measures are in place to take into account migrant issues to improve structure, processes, and outcome quality
Employee skills	Recognise and use staff with various backgrounds as resources
	(considered in recruitment)
Medical care procedures	Apply patient-centred approach, respect for patient autonomy
	Provide written processes regarding language assistance
Cross language communication	Define and regulate the use of various possible approaches to enable communication: external interpreters, immediate and telephone interpretation.
On-going employee training	Adapt form/content to needs
Central Services	Ensure administration, telephone services, enquiries, information, documentation, patient-related services (e.g., visiting rules), accommodation and catering, religious affairs and social services have knowledge and skills to address diverse needs
Network	Network with institutions

Main recommendations from “Diversity and Equity of Opportunity”, based on a brief summary p.9-11, Saladin, P. et al. [1].

As part of a follow-up strategy, a second Swiss MFH project [5] was initiated to create competence centres of MFH care. The federal government granted selected hospitals a total of CHF 2 milion to co-finance 50% of the pilot projects to develop MFH strategies (April 2010–April 2011) and their implementation (April 2011-June 2013). These projects were mandated to focus on trans-cultural competence of staff, use of professional trans-cultural translation and the improvement of the quality of health care for migrants. The document “Diversity and Equality of Opportunity [1] (see Table 1) was recommended as a guidance tool to develop these projects. The MFH project-quality criteria focused on project implementation and sustainability, with specific contents to be determined by hospitals. Five hospitals or hospital groups were selected based on their projects. One group, “AllKids”, was formed by three major, independent paediatric hospitals that applied together to fulfil the participation threshold of 2000 employees (Zurich, Basel, St Gallen). Two selected French-speaking university hospitals (Geneva and Lausanne) have major paediatric clinics attached. One consortium of two cantonal hospitals also has a paediatric service (Aarau). The remaining selected university hospital (Basel) provides only adult care [5].

### Hospital care for paediatric migrants – a topic of relevance?

For this research, we adopted a definition of migrants similar to that proposed by the International Organisation of Migration (IOM) in their background paper “Ensuring the right of migrant children to health care” [12]. This definition considers as a migrant person everybody outside of the territory of the state he/she is nationals of. It additionally considers all minors with a migrant background (second generation) as ‘migrant children’ independent of the child’s current nationality, thus taking into account that parental migrant experience and differences in language and culture will likely influence care approaches for children who were born in the host country and their parents. We will refer to minors with a migrant background as ‘paediatric migrants’ (PM) to distinguish them from adult migrants.

PMs represent a considerable group among the children served by paediatric hospitals. In Switzerland, for example, 23.3% of the inhabitants under 20 years [13] do not hold Swiss nationality –higher rates are found in some cities [14] – and many more share a migrant background.

Providing health care for paediatric migrants may be substantially different from that for adult migrants, and tools and concepts developed to improve care for migrant adults may need adaptation to the paediatric setting. In paediatrics, the patient-health professional relationship is transformed into a minor–parents/family–health professional-relationship. That is, providers must relate to both the patient and the guardians. This expanded patient approach suggests the need for specific service adaptations and professional guidance for the care of migrant youth and families. As far as we are aware, there has not been a comprehensive review or documentation of MFHs from a paediatric perspective, and current documents do not provide specific recommendations for paediatric hospitals [1,12,15].

**Figure 1:**
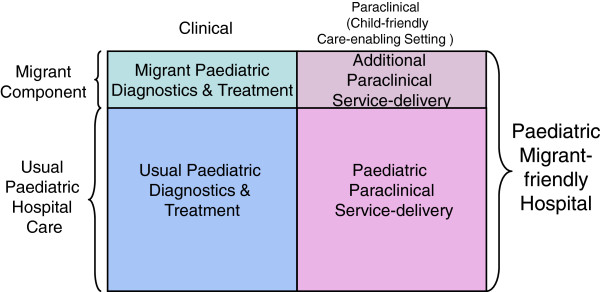
Paediatric Migrant-friendly Hospital components.

Optimal paediatric hospital care matches clinical excellence with child-friendly setting enabling care and recovery, taking into consideration the needs of the young patient and its family. This enabling setting is created through the paraclinical service delivery (e.g., adapted infrastructure, toys) component. Figure 1 illustrates that P-MFH-care may additionally require clinical competences in managing conditions linked to migration and disease patterns less common in non-migrant children, and that additional non-medical service delivery needs may have to be met (migrant component). The aim of this study is to explore the MFH concept, focusing on the non-medical service delivery (SD) aspects (services provided excluding the bio-medical aspects, including admission processes, accommodation, staff-care seeker interaction etc.) that are specific to the care of paediatric migrants.

In addition to an extensive review of the literature, we investigated experiences of professionals working at the five Swiss paediatric hospitals implementing the MFH concept. The objectives were to identify service delivery needs and challenges in caring for paediatric migrants and to explore differences in care compared to adult migrants.

It was not within the scope of this research to examine financial and legal access barriers (particularly renowned in countries such as the USA [16]) or to trace disease patterns associated with various migrant groups.

## Methods

### Interviews with project leaders of paediatric clinics participating in the MFH project

To explore practitioners’ perspectives and practice related to MFH service needs and implementation, semi-structured interviews were conducted with a purposively selected sample of staff members who were likely to be well informed about practices at the paediatric clinics participating in the MFH program in Switzerland.

All six paediatric clinics participating in the government MFH project were contacted by phone and email and invited to participate. One clinical director declined participation, explaining that his local project was not sufficiently advanced. For the other five participating hospitals, a semi-structured interview was conducted with a staff member responsible for MFH activities. Each staff member who participated held a high-level position in the MFH activities of his/her clinic, was part of the hospital decision-making hierarchy and had worked in migrant health for many years. However, their professional backgrounds varied: three were social workers, and two were senior physicians.

Consent forms and questionnaires were developed in English, translated into the local languages, French and German, and pretested with a senior medical physician to ensure the acceptability and relevance of the questions. Because study participants might attempt to present their hospital favourably, attention was paid to wording to reduce potential effects of social desirability. Interviews were conducted face-to-face in the language selected by the interview participant (1xFrench, 3xGerman, 1xEnglish) and at the interview location each participant selected. The average duration of the interviews was 1.5 hours. The interviews explored the service delivery needs of PMs and PMFs at their facility, challenges for staff, existing and anticipated approaches to meet needs, and participant’s perspectives on the current MFH strategies. Questions concerned the needs assessments conducted for the migrant-friendly hospital project and existing, planned or desired efforts to become more migrant-friendly. The questionnaire allowed for participants to freely identify needs, challenges, opportunities and interventions but also discussed more specific areas such as accommodation, food, family visits and sibling care, religious needs, interpretation services, sign posts and information material, relevant traditional health beliefs and acculturation, hospital processes, trainings, reference groups, migrant community involvement and a discussion on the feasibility of MFH interventions depending on the department size, from a paediatric perspective.

Interviews were transcribed and analysed in the respective language to avoid errors linked to translation. A “framework approach” [17] was chosen for analysis. This approach involved familiarizing oneself with the interview contents, establishing a topic framework that was then applied to the entire content. This indexing and the later charting (regrouping) were conducted using Nvivo8, which provided the basis for interpretation. Quotes, used to express current or opposed views, were translated to English by the interviewer, who is fluent in each of the languages.

### Literature search on non-medical service delivery components

To inform and complement the practice-related information collected through the interviews, an extensive review of the peer-reviewed literature on non-medical hospital service delivery needs of PMs and their families, on challenges to providers and on service approaches was conducted. Medline, Embase and Global Health were searched for relevant articles published since January 1, 1986, without language restriction. Intensive citation chasing was applied where appropriate (for key words and flow chart, see Additional file 1). This review was complemented by expert opinion.

The British National Health Service database, World Health Organization, IOM and UNICEF-webpages and http://www.mighealth.net were searched for relevant “good practice”, policies and guidelines. A general web search using a varying combination of search terms was performed, and the Swiss Federal Office of Public Health, Health Promoting Hospitals and European MFH task force were contacted by E-mail for additional information.

### Ethics

Ethics approval was obtained by the London School of Hygiene and Tropical Medicine ethics committee, and the contacted Swiss ethics authorities (e.g., Swiss Ethics, Kantonale Ethikkommission Zürich, Comission central d’éthique HUG) all confirmed that interviews with clinical staff did not require ethics approval in Switzerland. Clinic heads were informed of the research. Participants signed a consent form, decided on the level of confidentiality and were encouraged to state any alterations in the level of confidentiality they wanted observed during or after the interview, enabling them to give additional information that would not be quoted if desired.

## Results

Three social workers and two senior doctors participated as the representatives of migrant-friendly hospital activities of their hospital. All of them held senior positions. Participants were able to draw on their own experience and migrant care assessments conducted at their facilities. The three German-speaking paediatric clinics (together they form the “AllKids” cooperation group), for example, had previously conducted interviews with different categories of staff and parents in their hospital as a first step within their MFH project.

Four out of five clinics were affiliated with the local universities. All provide a wide range of paediatric and newborn, including intensive care, and have busy outpatient and emergency departments. The smallest and only non-university clinic participating, a secondary referral hospital, is the largest paediatric care provider in north-eastern Switzerland, still counting 3813 hospitalisations and more than 30 000 ambulatory contacts [18]. Geneva and Basel both account for more than 6 000, Zurich >7 000 and Lausanne >8 000 hospitalisations per year [19-21].

When asked to estimate the proportion of PM patients accessing their facility, interviewees from the French-speaking part of Switzerland estimated the highest numbers of PM patients (approximately 66-70% of hospital contacts). The lowest numbers (based on registered nationality) were reported in St.Gallen (~30%). Staff interviewed noted diverse patient backgrounds: e.g., Basel’s hospital representative suggested that the hospital treated 123 nationalities as out-patients and 97 as in-patients in 2009. These backgrounds also varied, with French-speaking hospitals attracting more migrants from French-speaking African countries and Latin American origin.

### Service needs

#### Paediatric migrants

When asked about PM needs, participants indicated that, because PMs are first and foremost children, many of their basic needs are the same as that of their non-migrant peers. One staff member noted the following:

Basically, all children have similar needs. They don’t want to stay at the hospital alone; they want their parents to stay, enough distraction and to go home healthy as soon as possible. (Social worker 3)

However, participants highlighted the greater importance of fostering migrant children’s sense of security and building trust:

The first need that pops up in my mind is safety: can we be trusted and are they safe where they are? (…)When they come to the hospital they are always afraid so we work hard … that they trust us and that they feel that we do care. This is for the children and the parents. (Clinician)

A participant explained that some PMs continuously move between two cultural value systems (home vs., e.g., school). At the hospital, PMs face the two systems simultaneously, which can create stress:

Children are torn between what they know from their parents and what they know from school/ kindergarten and what we offer here … Should I rather behave like Mummy and Daddy or like the nurses say. (Social worker 2)

When asked whether adapted toys, translated films and other means of distraction should be provided, opinions varied. While one participant, who often assists newly arrived or only transitioning children, considered having films and books in various languages and familiar toys a good idea, most others considered it unnecessary.

Children are children. I do not think they need other toys. Most go to Kindergarten and school here. (Social worker 3)

One hospital staff member mentioned a decorated room and African music for African children flown in by an NGO for operations but also did not consider further items necessary.

Citing an example of an African adolescent whose request for a priest had not been taken seriously by nurses, one participant underlined how religious needs in minors may be under-estimated because religion is often considered as separate from clinical care.

#### Parents

Respondents generally agreed that parents play a central role in paediatric care. Mutual understanding, information and a welcoming approach were identified as key needs of paediatric migrant parents (PMP) by all participants:

Parents need to feel that one wants them well, and this they sense, when one listens and tries to really understand them. (Social worker 2)

Needs of parents are: to understand and be understood, to be able to communicate actively with the medical personal and to know more about their child’s disease. (Social worker 1)

Language barriers make information less accessible for parents of PMs. According to participants, translated informational material was considered time saving, helpful and desirable by parents.

Several study participants noted that migrant families tend to have difficulties organising care for their healthy children at home when they wish to spend time with a hospitalised child. This difficulty may be accentuated by limited local family support or financial resources. Although some migrant groups (e.g., Northern European nationals) may have an above-average socio-economic status, many other migrant groups are among the lower socio-economic echelons [22].

Only one hospital occasionally allows siblings to stay overnight. Others sometimes try to use a subsidised charity hostel for parents and siblings. Lack of space in hospitals was given as a reason that siblings are not permitted overnight stays.

Staff also noted that parents may have achieved different levels of integration, even within the same family:

The father usually has been here for a while and speaks (…) well but the mother does not … for these people, we use and encourage the use of interpreters so that we can communicate directly with the parent. (Clinician)

All participants considered interpreters important to speak directly with parents who were less proficient in the local language.

One participant noted the greater need for privacy in some cultures. This is challenged when, for example, people must share a room with a person of the opposite sex or when breastfeeding. In paediatric hospital care, it is common to have one parent staying overnight. While a participant indicated that in most cases, it is the mother who stays, fathers staying over may result in non-sex-congruent parents spending the night in the same room. While one hospital stated that they work to redistribute patients to avoid men and women having to share a room, one interviewee had never heard that this issue was a problem. Another stated the following:

It is an issue in some cultures… in the context of hospitalisation not all needs can be met. We try our best… There are situations when it is impossible and parents need to accept this. (Social worker 1)

The staff member indicated that there are times when parents of migrant children may also need to compromise and adapt. Each participant stated that to improve privacy, all hospitals have rooms for breastfeeding and that whenever possible breastfeeding mothers receive single rooms in French-speaking hospitals.

#### Health professionals

Several study participants explained that health professionals need to work within certain service constraints. Several participants mentioned increased time requirements when caring for migrant children. One clinician even identified time as the most important challenge for physicians providing care for migrant families:

TIME. They need time. They need much more time than other kids because we have to understand their background; we have to understand not only their acute background but also their general background. It can be very puzzling, these family stories and the impact these stories have on their health and on their general standing. We have to take the time: just meet the child and meet the parents and try to understand what is happening but also to get to a translator and take time with a translator. Doing a personal history with an interpreter doubles the time – there is no doubt – and unfortunately that is what is mostly missing: time. (Clinician)

In addition to time requirements, staff reported that to work well and provide optimal care, they needed cultural knowledge on the meaning of diseases or family structures:

Staff’s desire and interest to gain more security when treating families with migrant background and to learn how to do better is great, …to know how the family system works, if there are different positions for girls and boys – once I know how the family system works then I know better how to approach them, what to consider, what is important. (Social worker 2)

A participant concluded that nurses appear to struggle more with immediate communication, while physicians are much more concerned about communicating diagnoses and the different understanding of diseases.

#### Interpreters

Most participants explained that interpreters needed to be familiar with the distinct paediatric disease vocabulary. This finding coincides with findings from the needs assessment by AllKids, which stated that translations of complex family structures, psychosocial problems, anorexia, obesity, AIDS etc. were considered very challenging by some interpreters. One participant noted the following:

When interpreting, there are topics where interpreters reach their limits and need training. (Social worker 3)

Interpreting in paediatrics can be emotionally very challenging. One participant illustrated this fact by highlighting the different meanings of chronic diseases or dying in a child or elderly. Several participants suggested that interpreters want to have briefings and debriefings before and after difficult interpreting sessions.

#### Hospitals

Paediatric hospitals (usually public or trusts) need to adhere to roles set by the political environment and maintain their budgets. Most participants expressed concerns about the hospitals’ ability to maintain future MFH activities in light of changing payment systems via diagnostic related groups (DRG) because this system does not allow additional remuneration for extra time spent, which was previously possible. Furthermore, interpreters are paid by hospitals, not patients. A social worker expressed hopes to be able to maintain activities via donations.

### Overarching challenges

#### Language

All participants agreed that language was a major challenge in providing P-MFH care – thus a challenge based on the common need for understanding and being understood shared by all individuals involved, including the child itself, too. All participants judged the use of professional interpreters as key, even if time consuming:

In the beginning we did not have a translator. It was horrible. Ok, it takes time, you always have to talk twice, but afterwards you gain so much. (Clinician)

A self-critical assessment conducted by a French-speaking hospital on parental language proficiency and its consequences revealed insufficient use of interpreters, even for important discussions, and a tendency to underestimate the need for interpreter assistance:

It was interesting to see that even people who sometimes did not speak French properly although they sort of manage when we talk to them directly, would need an interpreter. (French-speaking participant)

A common and very important difficulty reported was immediate or urgent interpretation, such as at the emergency department, which often is the main point of admittance to paediatric hospitals.

For emergency issues you have to deal with what you have. You won’t wait for two hours to have an interpreter to come in the middle of the night. (Clinician)

Frequently, interpreting cannot be done by a professional interpreter and instead relies on family or friends. Staff at each of the hospitals concurred that whenever possible, children should not interpret for their parents. High fees are a barrier to the use of phone interpretation. All participants reported using staff for ad hoc (i.e., immediate) interpretation but shared concerns about quality and bad experiences when using non-qualified staff. To avoid informal, ad hoc interpreting, one hospital tries to allocate their Spanish-speaking staff to consultations for Latin American undocumented migrants.

#### Intercultural challenges in paediatric care

Simply bridging the language gap was considered insufficient. Intercultural differences can lead to misunderstandings and additional challenges. A different understanding of roles and functions was given as an example:

Many migrant parents do not understand why they are supposed to participate in the medical decision process: They say: the physician has to decide, he is the expert! The only thing that counts is that the child gets well. (Social worker 3)

Similarly, in interviews by AllKids, some parents expressed difficulties with the Swiss approach to speak with and explain diagnoses or treatments to the child because they did not understand the right of a child to age-adapted information on her/his disease and treatment, which is considered important in Swiss paediatric clinics.

In several countries it is not common to directly address the child and speak with her/him in the parent’s presence. Many migrants asked us to speak with the head of the family. (Social worker 3)

Participants pointed to a learning process required by such parents. A similar learning process is also required for parental participation in care after discharge. As one participant explained:

It is not evident to many migrant parents, why when a child was treated here, they have to continue treatment at home… we have to work hard on compliance. (Social worker 3)

Adherence to care recommendations was therefore identified as a major challenge. A different understanding and acceptance of disease, particularly concerning disability, chronic conditions or psychosomatic problems, was thought to affect compliance and understanding.

The following quote suggests how staff’s knowledge, the use of trans-cultural mediators and specialised services can be useful to foster greater understanding:

We had a child who had big psychiatric problems and the parents were completely convinced that it was because the child was bewitched. They came from a small African village and were sure that something had happened there. Obviously, here people are not very aware of this thing and do not ask about it, so they (staff) just thought that parents were against psychiatric care or they just laughed about it – but this is very serious business for the family – it means a lot, it is their whole believe – so the ethno-psychiatric consult helps us to take care of these children taking into account the background of the child and the parents. (Clinician)

Culturally appropriate group teaching at the hospital, e.g., on diabetes, was also considered helpful.

### Not just small adults – differences to adult MFH care

The interviews highlighted participants’ opinions about the aspects of paediatric care that are likely to differ from adult care, which must be considered in developing paediatric MFH- services. Aspects identified to most likely differ in the provision of adult and paediatric care included the following:

•Different disease patterns (paediatric diseases)

•Admission often via emergency room, shorter hospitalisations, often immediate decisions required

•More individuals involved in care planning

•Primary decisions & responsibility by family, (usually) not patient

•History often told by parents, not child patient

•Care (at the hospital and after discharge) usually impossible without the family

•Different levels of language-skills and acculturation of minor, mother and father: different resulting needs/views

•Different position/meaning of a sick child vs. an adult in the family

•Hours of consultation not only determined by child but also by the parents’ availability to bring the child to the facility

•Care for siblings may be a concern

•Rooming-in (Room-allocation based on gender of child or parent staying?)

•Child in the middle of development, different vulnerabilities

•Child’s thinking focuses on present

•Legal matters, truth of story not of primary interest

### Approaches chosen by hospitals

Based on different needs, different approaches are currently used to address care challenges that are specific to PMs, e.g., various forms of interpreter services, trans-cultural mediators, training, specialised consultations, and food catering adapted to religious needs.

#### General approach

One participant emphasised the importance of adopting an integrative, welcoming approach, focusing on everyday processes, individual needs and adaptations benefiting all, not only migrant families.

We emphasise that the nurses – all in their own way - talk with the mothers about things that are important to her [mother]: the name’s meaning, rituals, and from there you can start. Before, it was the checklist: the bath, the breast, (…) Now, the fact of being attentive is often enough..... It’s for everyone! (Clinician1)

During the morning debriefing of all teams, a standard question is: is there a communication problem? If yes, we order an interpreter. (Clinician)

One university hospital has a separate consultation for asylum seekers also caring for undocumented migrants, if needed.

#### Staff and training

Staff members’ varied backgrounds are considered a resource in providing P-MFH care, whether it is because of their own personal experience of migration, language knowledge or intercultural experiences. Positive attitudes and knowledge about P-MFH care were considered key; however, currently, training on how to assist PMs varies from no training for some to clinical case discussions for junior physicians and 1,5-day trainings mainly for nurses (physicians lack time). Three hospitals train junior staff regularly in the use of interpreters. Clinicians emphasised the importance of training in patient-history-taking skills considering migrant needs. Training modules offered for adult and child health care providers together were discussed. Because generic modules often focus on challenges mainly encountered in adult health care but do not sufficiently consider paediatric aspects, they were considered less useful.

The adult population is much more worried about legal stuff; about Post-Traumatic-Stress-Disorder; about true stories or untrue stories. There are different things that are more important for adults than for children. We don’t really ask them to tell their stories, how they felt it but how they are feeling now. Children are much more in the present than in the past. And the parents, we use the parents much more to tell us what has happened. (Clinician2)

All participants considered that an interdisciplinary reference group involving all staff levels is essential to promote further migrant-friendly hospital development and staff support.

#### Infrastructural and service adaptations

Infrastructure is often limiting and difficult to change. The construction of a new building was used as an opportunity to improve the migrant friendliness of their infrastructure (multi-faith room, space for numerous visitors) by one clinic.

The importance of adapting the building structure or decor was suggested by one participant who explained that colour/picture codes and signposts in multiple languages should be used. In contrast, another participant disagreed and considered the existing pictograms and wording in the local language sufficient, explaining that everybody understood “emergency”.

Alongside the previously mentioned services, all participants emphasised the importance of social services to help organise care for healthy siblings, health insurance for undocumented migrants, or financial assistance.

#### Migrant involvement

AllKids tried to involve migrant communities by interviewing parents of PMs during their needs assessment and hoped to increase the currently insufficient feedback for quality control, e.g., by announcing feedback possibilities in various languages. Feedback difficulties were also explained culturally:

In many countries, it isn’t common to be asked about your opinion as a patient. (Social worker 1)

Interpreters from migrant communities were considered potential representatives and partners.

### The Swiss MFH project and MFH criteria

Several participants mentioned difficulties linked to the lack of clear criteria and standards. In terms of satisfaction with the Swiss MFH project implementation in regards to collaboration among partners, high satisfaction was expressed among participants of purely paediatric projects. Levels of satisfaction varied greatly among paediatric partners in adult-health-care driven projects where reduced access to decision making and funding was expressed as a concern. One participant considered reduced decision power and funding to be a logical consequence of paediatric departments being smaller than adult care while another one questioned the MFH project’s potential to impact paediatric care due to lack of involvement and funding.

### Literature on non-medical Service Delivery Needs and Approaches

Primary research on non-medical hospital SD needs and on approaches concerning PM hospital care appears limited. Many articles on PMs discuss limited access to healthcare for financial or legal reasons in the US [16], some report health outcomes and fewer discuss cultural child health ideas [23-25].

Among more than 2500 search hits, 226 were identified as potentially contributing to a better understanding of migrant paediatric service delivery needs and approaches in the widest sense, using a very generous inclusion approach. Among 72 articles potentially dealing with service delivery for migrant children, 55 could be retrieved for a full text assessment. Intensive reference chasing and assessments of the 55 full text articles allowed identification of only six quantitative hospital-based studies on SD-needs and approaches (See Additional file 1 for flow chart).

A US trial on interpreters at a paediatric emergency room (ER) [26] adjusting for disease severity showed significantly higher rates in the use of diagnostics, IV fluids, in hospitalisations as well as higher costs in language-incongruent compared to language-congruent encounters. While hospitalisation rates remained (less) high (OR=1.7; 95%CI 1.1-2.8), costs, IV fluid use and testing were reduced to the level found in natives using interpreters; however, consultation time increased by +16 (95%CI 6.2-26) minutes. Interpreter allocation may have been influenced by staff; socio-economic factors were not considered.

A trial, randomly allocating interpreters [27] for language-incongruent ER encounters, found increased satisfaction with physicians’ performance in parents who had access to hospital-trained interpreters compared to ad hoc interpretation by staff or relatives.

A short training intervention in paediatric residents [28] on the availability of a new program for migrants showed effects on referral practises even after four weeks. The selection of participants, who often had a migrant background, was not explained. A trial assessing the effects of Spanish-language training for doctors improved the relationships with physicians from the perspective of PMP [29].

A quantitative study on 129 Italian hospital nurses identified language, diet (54%), hygiene (51%), different pain perception (44.9%) and facilitating religious needs as problematic. The response rate was only 64% [30]. In a similar study, again suffering from a low participation rate (37.2%), language, extended family, non-compliance, religious customs and culturally inappropriate food and nursing gender roles were mentioned as problematic [31]. The external validity of these studies may be limited as the local setting and migrant groups served vary.

Two additional paediatric Swedish primary care studies are of potential interest for out-patient hospital care:

In a survey among 270 nurses, these nurses state feelings of inadequate cultural knowledge, lack of direct communication with the family and worries about health advise not being understood (>90% of nurses), incorrect interpretations (71%) and time constraints (50%) as concerns. A randomised controlled trial evaluating trans-cultural training in nurses [32] showed no or only little effect in most assessed points. The study suffered of low power, (24 in intervention group) and recruitment difficulties possibly affected the results.

Qualitative research and overview articles further mention time constraints and challenges to communication: Non-language-proficient parents - dependent of interpreters – can see their statements commented by language proficient PMs before complete interpretation by the professional [33]; lack of direct communication during interventions can be stressful for nurses and fearful for children [34]; and desired information flows between parents may vary and in some cultures be controlled by fathers for mothers and children [34]. Most studies fail to identify the migrant population served, bearing the risk of undue generalisations.

No further guidelines for paediatric hospitals to become migrant-friendly were found.

## Discussion

### Paediatric non-medical service delivery needs

This research identified important core non-medical service delivery needs and challenges associated with the care of PMs encountered by those mandated to participate in Switzerland’s migrant-friendly hospital care programme. Results indicate that key actors (PMs, different family members, different health care staff, interpreters, hospitals/management, society) all have individual needs (see Additional file 2 for summary of individual needs) but face common overarching challenges (language barriers, cultural differences, service constraints, etc.) in the pursuit of appropriate service delivery to children and their families.

International research on paediatric migrant services is scarce and focuses mainly on nurses [30-32,35], resulting in an evidence gap on the needs of other involved actors. Nurses’ difficulties described in the literature were mostly related to language, compliance, culture, family involvement, and diet [30,31,35]. Aside from concerns about hygiene and culturally appropriate diet, which were not raised specifically by respondents in our study, a majority of our findings are aligned with most previous findings; however, we also identified additional needs specific to PMs, the family, and hospital staff, such as security, a welcoming approach or care of healthy siblings.

Literature and interviews highlighted language barriers and cultural differences as key challenges [30,31,34,35]. Time constraints [36] may further exacerbate these challenges. If these issues remain poorly addressed, they may result in misdiagnoses and noncompliance leading to poorer patient health and increased cost to the health sector.

Some of the needs identified, such as information and a welcoming approach, are similar to those in adult care. Other needs are specific to paediatrics (e.g., consideration of different levels of acculturation/language skills of PMs, their parents or guardians, and care of siblings). Differences to adult care indicate that generic MFH recommendations may inform approaches (training, translation); however, the implementation requires adaptation to paediatrics (e.g., training contents).

Many needs described in interviews, such as care for healthy siblings, intimacy, information, respect of health beliefs, a receptive environment and time are potentially more visible or marked in certain PM families but are also shared by native families [37]. Revising responses to PM families and mainstreaming [15] may therefore be beneficial to all families, increase acceptance of the MFH approach in the local population and reduce the risk of stigmatisation.

Needs of staff and translators, such as for emotional support and training, were also identified. If staff needs, particularly affected by time constraints, language barriers, feelings of insecurity (worries about poor health outcomes due to cultural/language misunderstandings) [35], remain poorly addressed, these may evolve into negative feelings about their work and the patients [38].

### Defining P-MFH

Interviews illustrate a broad range of approaches to meet the needs of all involved: e.g., interpreters, cultural-mediators, and training, diet and infrastructure adaptations.

Criteria and standards [39] for P-MFH are still to be defined. Diverging opinions of our participants regarding potential migrant-friendly adaptations, such as special toys, multi-language sign posts, accommodating siblings or matching same-sex parents staying overnight, illustrate that recognising needs and the willingness to address them depends on information, feasibility, values and experiences, the migrant population served and the level of acculturation efforts expected from the migrant population. Identification of criteria and the setting of achievable and measurable standards have a socio-political dimension.

The choice of P-MFH-defining criteria should consider research evidence on approaches for providing migrant-friendly hospital services. Relevant research focusing on the paediatric setting is still scarce, of variable quality and predominantly focuses on Latino populations in the USA [26-29], indicating further research needs in Europe. Transferability of these US studies may be limited due to the difference in settings. Medical language classes for motivated physicians with basic foreign language skills may be useful when the migrant groups are homogeneous [29] but less feasible when multiple languages need to be addressed.

The use of interpreters had been considered key by respondents and has previously been validated in paediatrics [26,27]. It has cost-saving potential and reduces rates of hospitalisation and medical interventions in paediatrics [26,40]. Flores et al. demonstrate the non-negligible health risk of poor quality interpretations – by professionals or family members- in paediatrics (e.g., when important information such as allergies are omitted by the interpreting person) [41]. Due to its direct impact on health outcomes, high quality interpreting should be a priority.

Trans-cultural training [1,7,15,42] was evaluated beneficial in adult care and important by participants. Training needs to be adapted to the specific paediatric audience, considering time constraints. In the paediatric setting, studies are not yet conclusive [28,32].

Evidence on the benefit of further adaptations still needs to be gathered.

**Figure 2:**
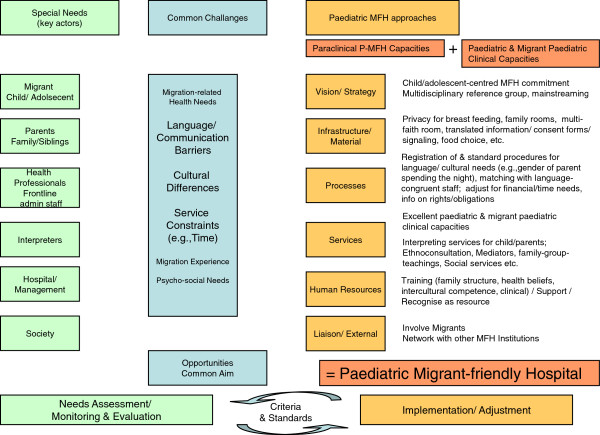
Needs-based Approach to Paediatric Migrant-friendly Hospitals (P-MFH).

Considering the differences in PM populations served and hospital resources, hospitals currently need to define their own strategy. Striving for P-MFH involves reflecting on the needs of key actors (e.g., PMs) and common challenges, setting priorities and evaluating the feasibility of changes to hospital processes and structures [43]. Figure 2 illustrates the main key actors whose different needs translate into challenges that need to be addressed in order to provide migrant-friendly hospital care. It further demonstrates the various domains where a MFH approach may be beneficial. Continuous self-critical monitoring and evaluation followed by adaptations feeding into a quality improvement cycle [39] should consider reviewing standards, progressively increasing them. Staff with their diverse background and willingness to learn and migrant families can be a resource to attain the common aim: PMs’ health.

Obviously, in order to be truly migrant-friendly, the clinical part of care also needs to be up to date with PM health issues [44].

### The Swiss MFH pilot project and policies

The MFH pilot project is a catalyst for P-MFH. Because many paediatric units are part of bigger hospitals and none have 2000 staff, their participation was limited. Local co-operation is necessary and useful; however, the project also demonstrates that if solutions are not paediatric driven, they risk to neglect paediatrics. Small paediatric units do not have the resources, numbers of PM families and structural possibilities to implement all ideal P-MFH approaches but may benefit from an exchange of resources, such as paediatric-specific MFH-care training, consent forms or brochures, with other paediatric departments.

The project aimed at creating competence centres [5]. While the lack of clear criteria may have the advantage of keeping possibilities open for innovative approaches adapted to local needs, a minimal set of standards to be met may be useful to commit and motivate hospitals.

The feasibility of MFH implementation can be jeopardised by financial and socio-political constraints, threatening consultation time and interpreter use. To guarantee sustainability of current efforts, the Swiss government will have to carefully monitor the negative impacts of the new payment system (Swiss-DRG), which pays hospitals on the basis of retained diagnoses not intending to cover additional time needs required for MFH efforts or interpreter costs. International experiences also demonstrate that policies may affect health care staff’s ability and satisfaction to provide migrant-friendly care [45].

### Limitations

Hospitals serving smaller migrant communities and small paediatric units were not interviewed. In addition to organisational challenges linked to size (e.g., special services for small numbers are less affordable), the main challenges are likely to be similar and the recommendations are only helpful within the means of the departments. No interviews with parents of PMs, PMs, nurses, administrators or junior physicians were conducted. This limitation may have partly been compensated by interviews with key staff who offered their observations based on their experience with migrant-friendly activities in their institutions, particularly the needs assessments conducted for the MFH projects. The survey by AllKids e.g., on which German-speaking participants based some of their responses, at least captured the PMP views of selected migrant groups (e.g., Turkish, Albanian, and Tamil) in three regions and of various staff members, particularly nurses. Not interviewing nurses may have been compensated by triangulation with the international literature, which focuses on their needs [30,31,35].

While the number of interviews conducted was relatively small, by selecting participants because of their central role in investigating migrant child health issues for the MFH project, these individuals are able to offer a unique overview about a range of issues concerning migrant child healthcare. The priorities mentioned across this small cohort remained consistent strengthening their validity. While the small number of interviews reflects the small number of paediatric clinics involved in the migrant-friendly hospital project, the fact that five out of six paediatric clinics were ready to participate, allowed best possible coverage of migrant-friendly project hospitals.

Needs and approaches identified in interviews are relevant for Switzerland and its migrant population. International generalisations need precautions even though triangulation of evidence from interviews with existing literature strengthened the analysis. Recommendations, particularly those for hospitals, are relevant for Switzerland; however, concepts may be adapted with caution to other European settings.

Because the subject is very broad with many subcomponents (e.g., training, needs of mothers and interpreters) and a standard terminology still missing, a broad approach had been chosen to cover as much relevant information as possible. Although a large number of articles were screened and many references chased, it is not possible to be certain that all relevant articles were identified.

### Recommendations

Recommendations are based on the present research including existing recommendations on MFH and paediatric migrant health care [1,7,12,15].

•Define a clear P-MFH vision and strategy and minimum standards;

•Identify a reference team composed of different professionals, including senior management and frontline professionals to ensure the implementation of PM care that is congruent with everyday reality.

•Develop a P-MFH care strategy based on an evaluation of:

○ Needs of PMs, parents and siblings, human resources, interpreters;

○ Key challenges: language/cultural differences, time limitations, acculturation levels of PM/-parents;

○ Opportunities (e.g., human resources’ variety and motivation, migrant families and interpreters).

•Revise hospital processes from admission to discharge [1] tailored to the needs of migrant children and their families (e.g., register different language proficiency levels/acculturation levels, possibly matching with language congruent staff, colour coded sign posts)

•Revise structural components [43] related to: infrastructure (privacy for breastfeeding, space for visitors/siblings); services (social services, trans-cultural-mediators, easily available interpreting services, contacts for religious/language congruent support); human resources.

•Address language barriers to avoid potential harm and costs; make immediate translations available for parents and PMs, e.g., phone-interpretation.

•Recognise staffs’ diverse backgrounds and motivation as a resource.

•Provide staff support and skill training in intercultural and paediatric migrant health care (e.g., on PM health, child health beliefs, family structures etc.)

•Recognise staffs’ migrant-friendly efforts, e.g., longer consultations due to the need for translations, even if it increases time requirements.

•Consider innovative approaches to solve problems (e.g., family rooms to allow sibling to stay over).

•Involve partners (migrant groups, other hospitals, etc.)

•Avoid stigmatising

•Conduct regular evaluations of migrant friendliness while continuously raising standards.

•Use migrant-friendly health aspects to reflect on emerging care needs for migrant and non-migrant children and families alike and mainstream solutions

Various service providers, including migrant organisations, can also contribute to improving the migrant friendliness of paediatric hospital care and to setting minimal standards. Paediatric bodies may offer short courses on paediatric migrant health issues and become involved in the formulation of recommendations and policies. Further research should evaluate the needs not only of nurses but also of PMs, their families and paediatricians and evaluate P-MFH approaches. It should recognise different migrant groups to ensure comparability and to avoid masking potential health service needs. Stigmatising needs to be avoided. Platforms to exchange P-MFH ideas and resources may increase collaboration (e.g., http://www.mighealth.net, HPH, paediatric hospitals working on improving migrant health, and paediatric bodies).

Future Migrant-friendly health policies and programs should account for the special needs of PMs and consider the specific concerns of paediatric clinics linked to often smaller, dependent department structures and facilitate the exchange between them. Setting minimal standards of paediatric MFH care may be useful.

To ensure that MFH policies are sustainable, the government needs to ensure feasibility and avoid potentially conflicting policies, as well as monitor possible side effects.

Needs assessments, developing care approaches and delivering care for PMs should be recognised as an opportunity to reflect on our health services, health policy trends, and needs as well as a chance to further improve health services for all – migrants and non-migrants.

## Conclusion

To provide care that is tailored to the needs of migrant children, hospitals will have to consider the specific service needs of PMs and families, staff and external interpreters. Minors are not simply small adults. While overarching approaches, concepts and categories described in Diversity and Equality of Opportunity [1], IOM-materials [12,15] and the MFH Amsterdam declaration [7] can be useful, they require adaptation to be fully implemented in paediatric care settings. International research on hospital care for migrant children is scarce and should be intensified. Recommendations to meet the needs of paediatric migrants and their families may differ from guidance for adults. Paediatric needs should be systematically identified and the findings should be used to inform paediatric hospital care approaches. Hospital processes from admission to discharge should be revised to ensure implementation of migrant-sensitive approaches suitable for children. Staff should receive adequate support, such as training, easily available interpreters and sufficient consultation time, to be able to provide migrant-friendly paediatric services. Involvement of migrant groups may be helpful. Improving quality of care for PMs at the policy and service levels is an investment in the future that will benefit native and migrant families.

## Abbreviations

MFH: Migrant-friendly hospitals; PM: Paediatric migrants (migrants younger than 18 years); PMP: Paediatric migrant parents; P-MFH: Paediatric migrant-friendly hospitals.

## Competing interests

The authors’ declare that they have no competing interests.

## Authors’ contributions

FJ developed the research idea and study design, conducted the interviews, literature review, analysis and interpretation of findings, and drafted the manuscript. CZ participated in the study design, interpretation of data and critical review of the manuscript. LK and MH contributed through inputs during the study period and a critical review of the manuscript. Everyone gave approval for publication. All authors read and approved the final manuscript.

## Pre-publication history

The pre-publication history for this paper can be accessed here:

http://www.biomedcentral.com/1472-6963/13/389/prepub

## Supplementary Material

Additional file 1 Academic literature search. Click here for file

Additional file 2 Identified needs (selection). Click here for file
