# Supplementary material for: Migrant-friendly hospitals: a paediatric perspective - improving hospital care for migrant children
Source: BMC Health Serv Res. 2013 Oct 5;13:389. doi: 10.1186/1472-6963-13-389 (PMC3852418; doi:10.1186/1472-6963-13-389)
Supplement: Additional file 1 — Academic literature search. [file 1472-6963-13-389-S1.docx]

Additional file 1:

Academic literature search:

1. Keywords: child or children or minor* or adolescent* or boy* or girl* or teens or

teenage* or youth* or underage* or juvenile* or paediatric* or pediatric* or young

person* or young people or toddler

2. Subject headings: (adolescent/ or child/ or child, preschool/ or infant/)

3. 1 or 2

4. Keywords: (migrant* or immigrant* or migrant*OR refugee* or asylum-seeker or

asylum seeking)

5. Subject heading: (Transients and Migrants/)

6. 4 or 5

7. Subject heading: (Delivery of Health Care/) or Keyword: health care.

8. Keywords: (Service* adj3 delivery) or polic* or guideline* or transcultural or

translation

9. 7 or 8

10. 9 and 6 and 3

11. 10 limited: 1986 to current (July 2011) and paediatric age.


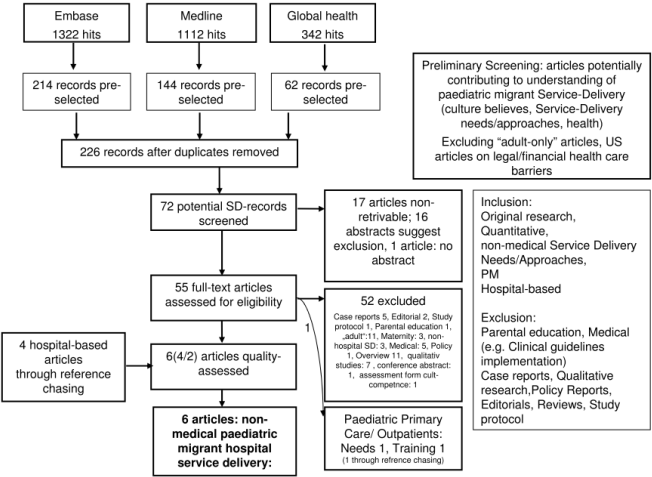


Two primary health care based articles were additionally analysed for bias and confounding, as they seemed relevant.

2. Search: Embase Keywords: migrant-friendly hospitals OR migrant friendly hospitals: 1

non-paediatric hit
