# Supplementary material for: Migrant-friendly hospitals: a paediatric perspective - improving hospital care for migrant children
Source: BMC Health Serv Res. 2013 Oct 5;13:389. doi: 10.1186/1472-6963-13-389 (PMC3852418; doi:10.1186/1472-6963-13-389)
Supplement: Additional file 2 — Identified needs (selection). [file 1472-6963-13-389-S2.docx]

Additional file 2:

**Identified needs (selection)**

| Migrant child/adolescent | - Combined excellent conventional and migrant paediatric diagnostics and treatment (somatic/psychological) - Age-appropriate information/communication - Age-appropriate occupation (toys, schooling, etc.) - Security, trust (parents present) - Food they like - Consideration of level of acculturation - Consideration of migration experience - Religious/Cultural needs |
| --- | --- |
| Parents/Family/Siblings | - Know their child receives good care - Appropriate information (incl. written)/ communication and consideration of individual levels of language proficiency - Consideration of level of acculturation of different family members - Care of healthy siblings - Respect/empowerment/security - Consideration of migration experience - Religious/Cultural needs |
| Health professionals incl. frontline admin staff | - Able to do their work - Training & support - Time - Respect and empowerment/security - Communication/ Information |
| Interpreters | - Training & support |
| Hospital/Management | - Comply with socio-political environment/pressures - Finance/Budget in line - Good reputation/patient satisfaction |
| Society | - Comprehensive healthcare according to resources/needs |
